# The ginger polyphenol 6-gingerol elicits minimal changes in an *ex vivo* human gut microbiome

**DOI:** 10.3389/fnut.2025.1711783

**Published:** 2026-01-26

**Authors:** Karley K. Mahalak, Adrienne B. Narrowe, Jenni Firrman, Johanna M. S. Lemons, LinShu Liu

**Affiliations:** Dairy and Functional Foods Research Unit (USDA), Eastern Regional Research Center, Agricultural Research Service, United States Department of Agriculture, Wyndmoor, PA, United States

**Keywords:** 6-gingerol, ginger, gut microbiome, polyphenols, short-chain fatty acids (SCFAs)

## Abstract

Ginger (*Zingiber officinale*) has a long history of use in traditional medicine and the modern world to alleviate health conditions, particularly those related to indigestion and nausea. Gingerols are phenolic bioactive compounds found in ginger. It has been suggested that health benefits associated with gingerols may be due to modification of the gut microbiome, especially in disease models. However, the impact of gingerol on a healthy human gut microbiome, and whether age affects gingerol activity, is not well understood. To address this, the impact of 6-gingerol, the most abundant polyphenol found in ginger, on the gut microbiomes of four age groups (infants, children, adults (22–40), and adults (60+)) was determined using SIFR® technology. Following a 24-h incubation with 6-gingerol, microbial community genomic analysis was performed together with metabolic analysis to determine the impact of 6-gingerol on the gut microbiota *ex vivo*. Using this method, 6-gingerol was determined to have no significant impact on the gut microbiota in terms of community density, community diversity, or short-chain fatty acid production. This study found that, in healthy gut microbiota, 6-gingerol did not have a strong effect within a 24-h period of treatment.

## Introduction

The gut microbiota is a complex community of organisms that changes and develops throughout life in response to various stimuli, including hormonal shifts, environmental changes, and activity level (1). However, one of the main mechanisms for modification of the gut microbiota is through dietary alterations (2, 3). Intentional and unintentional alteration of the gut microbiota through dietary intake has been a major focus of research on gut health. Many major dietary modifications cause significant changes to the gut microbiota in terms of its community structure and functional output within as little as 24–48 h (4).

The impact of gut microbial community structure and its functional outputs, on not only gut health but overall health, has resulted in increased interest by both industry producers and consumers to identify dietary changes and to develop supplements that may improve health outcomes. These dietary changes and supplements often include probiotics (live bacterial strains) and prebiotics (dietary fibers) but may also include other small molecules found in fruits, vegetables, and herbs, such as polyphenols (5). Following consumption, the majority of polyphenols (>90%) are unchanged through the digestive system until they reach the colon (6, 7). Upon reaching the colon, the gut microbiota may degrade the polyphenols into intermediary and end products, which have increased bioavailability as compared to the original polyphenols, and their subsequent absorption may be the cause of some health benefits associated with polyphenol consumption (5, 8).

Ginger (*Zingiber officinale*) has a long history of use as a traditional medicine, particularly in the form of herbal tea, which is a major contributor of dietary polyphenols (9). For gastrointestinal health in particular, ginger is often used to alleviate nausea, vomiting, and indigestion (10). Powdered ginger root has also demonstrated effectiveness against gastrointestinal disorders and disease in colitis mouse models by reducing inflammation and modifying the gut microbiome structure (11). There are many different bioactive compounds in ginger, including its polysaccharides, which have been shown to alleviate intestinal inflammation and help to remediate changes to the gut microbiome that occur in colitis mouse models (12). Ginger also contains multiple polyphenols, including: 6-gingerol, 8-gingerol, 10-gingerol, 12-gingerol, and 6-shoagal. Of these, the most abundant is 6-gingerol (13). Previous work using *in vitro* fermentation of mouse feces found that incubation with an extracted polyphenol mixture from ginger modified the gut microbiota by increasing the abundance of Firmicutes and Actinobacteria, and increased the production of short-chain fatty acids (SCFAs) (13).

6-gingerol has shown promise in benefiting health in a number of disease states, including anti-inflammatory and antioxidant activity (14, 15). 6-gingerol has also demonstrated efficacy in anti-obesity effects, including modification of the gut microbiota in mice fed a high-fat diet (15). Like many polyphenols, 6-gingerol largely reaches the colon intact and is then transformed by members of the gut microbiota into intermediary and end-products. 6-gingerol is degraded through glycosylation by glycosyltransferases (GT), and specifically by uridine diphosphate-glucose (UDP)-GTs (16). However, little has been done on the impact of 6-gingerol on a healthy gut microbiota, or across age groups.

To address this research gap, we aimed to outline the impact of 6-gingerol on the healthy human gut microbiota across age groups, including breast-fed (BF) infants, children, adults 22–40, and adults over 60 using *ex vivo* Systematic Intestinal Fermentation Research (SIFR®) technology. DNA sequencing and metabolomic analysis were performed after incubation for 24-h to determine whether 6-gingerol meaningfully modifies the healthy gut microbiota in terms of either structure of function.

## Methods

### *Ex vivo* fermentation using SIFR®

*Ex vivo* fermentations were performed by Cryptobiotix (Belgium) using SIFR® technology, similarly to those described previously (17). Fecal samples were collected from 9 healthy donors from each age category: Breastfed infants (0–3 months)(BF infants), children (5–8 years old), adults 22–40 years old, and adults 60+ years old. Fecal collection was performed according to IRB approval from the Ethics committee of the University Hospital Ghent, Belgium (BC-09977). Exclusion criteria included no probiotic, prebiotic, or antibiotic use for 3 months prior to donation. Fermentations were performed for 24 h. Two fermentations were performed for each donor, one with 1.2 mg/L 6-gingerol (Bio-connect, Huissen, The Netherlands) treatment, and one that was the no-substrate control (NSC). Medium M0019 (Cryptobiotix, Ghent, Belgium) was used for fermentation for BF infant donors, M0017 (Cryptobiotix, Ghent, Belgium) was used for fermentations for children and adults.

### DNA extraction, library preparation, and 16s rRNA gene sequencing

DNA extraction was performed as described previously using a SPINeasyDNA kit for Soil (BP Biomedicals, Eschwege, Germany) according to the standard instructions (18).

Genomic sequencing was performed targeting the V3-V4 region of the 16S rRNA gene using 2×300 bp chemistry on an Illumina Miseq (Illumina, San Diego, CA) following the manufacturer’s guidelines (19). Briefly, primers (Integrated DNA technologies, Coralville, IA) amplified the 16S variable region using 2X KAPA HiFi HotStart Ready Mix (Fisher Scientific, Hampton, NH) and AMP XP beads (VWR International, Radnor, PA) were used for PCR clean-up. Indexed libraries were generated using NExteraXT Indexes (Ilumina, San Diego, CA). Libraries were combined with 30% PhiX (Illumina) as an internal control and 10pM loaded on a V3 reagent cartridge (Illumina).

Total bacterial cell counts were determined using a BD FACS Verse flow cytometer (BD, Erembodegem, Belgium) as described previously and analyzed using FlowJo V. 10.8.1 (20).

### GC-FID analysis of short-chain fatty acids and other metabolic products

SCFA (acetate, propionate, butyrate and valerate) and branched-chain fatty acids (bCFA; sum of isobutyrate, isocaproate and isovalerate) were determined via gas chromatography with flame ionization detection, upon diethyl ether extraction, as previously described (21). Briefly, 0.5 mL samples were diluted in distilled water (1:3), acidified with 0.5 mL of 48% sulfuric acid, after which an excess of sodium chloride was added along with 0.2 mL of internal standard (2-methylhexanoic acid) and 2 mL of diethyl ether. Upon homogenization and separation of the water and diethyl ether layer, diethyl ether extracts were collected and analysed using a Trace 1,300 chromatograph (Thermo Fisher Scientific, Merelbeke, Belgium) equipped with a Stabilwax-DA capillary GC column, a flame ionization detector, and a split injector using nitrogen gas as the carrier and makeup gas. The injection volume was 1 μL and the temperature profile was set from 110 °C to 240 °C. The carrier gas was nitrogen, and the temperatures of the injector and detector were 240 and 250 °C, respectively. pH was measured using an electrode (Hannah Instruments Edge HI2002, Temse, Belgium). Lactate was quantified using an enzymatic method (Enzytec™, R-Biopharm, Darmstadt, Germany).

### Bioinformatics and statistical analysis

Raw reads were processed using DADA2 (22) within the QIIME2 (v 2–23.7) environment (23) with the parameters p-trim-left and p-trim-right = 15; p-trunc-len-f and p-trunc-len-r = 250. ASVs were taxonomically classified against the Silva 138–99 database (24) using a naïve Bayes classifier implemented in scikit-bio (25). A rooted phylogenetic tree was generated using MAFFR and weighted UniFrac distanced calculated. Principle coordinates analysis was implemented using R library ‘ape’ (26) and visualized using tidyverse (27).

Community functional prediction was performed using PICRUSt2 (v.2.5.1) (28). Tests for significantly differentially abundant taxa (phylum and genus level), and functions were performed using MaAsLin3 (29) treatment was used as the fixed effect with door as the random effect. PERMANOVA testing for significant clustering by treatment across all ages or within age groups was performed using pairwise.adonis.2 (30).

## Results

SIFR® technology was used to perform 24-h incubations with 1.2 mg/L 6-gingerol to determine the impact of the polyphenol on the gut microbiome of 4 separate age groups in terms of community structure, relative abundance, and fermentation products. This dose was selected based on the amount of 6-gingerol found in ginger extract (13) and previous work performed that determined 4 g/D of ginger consumption was able to reduce chronic disease (31).

**Figure 1:**
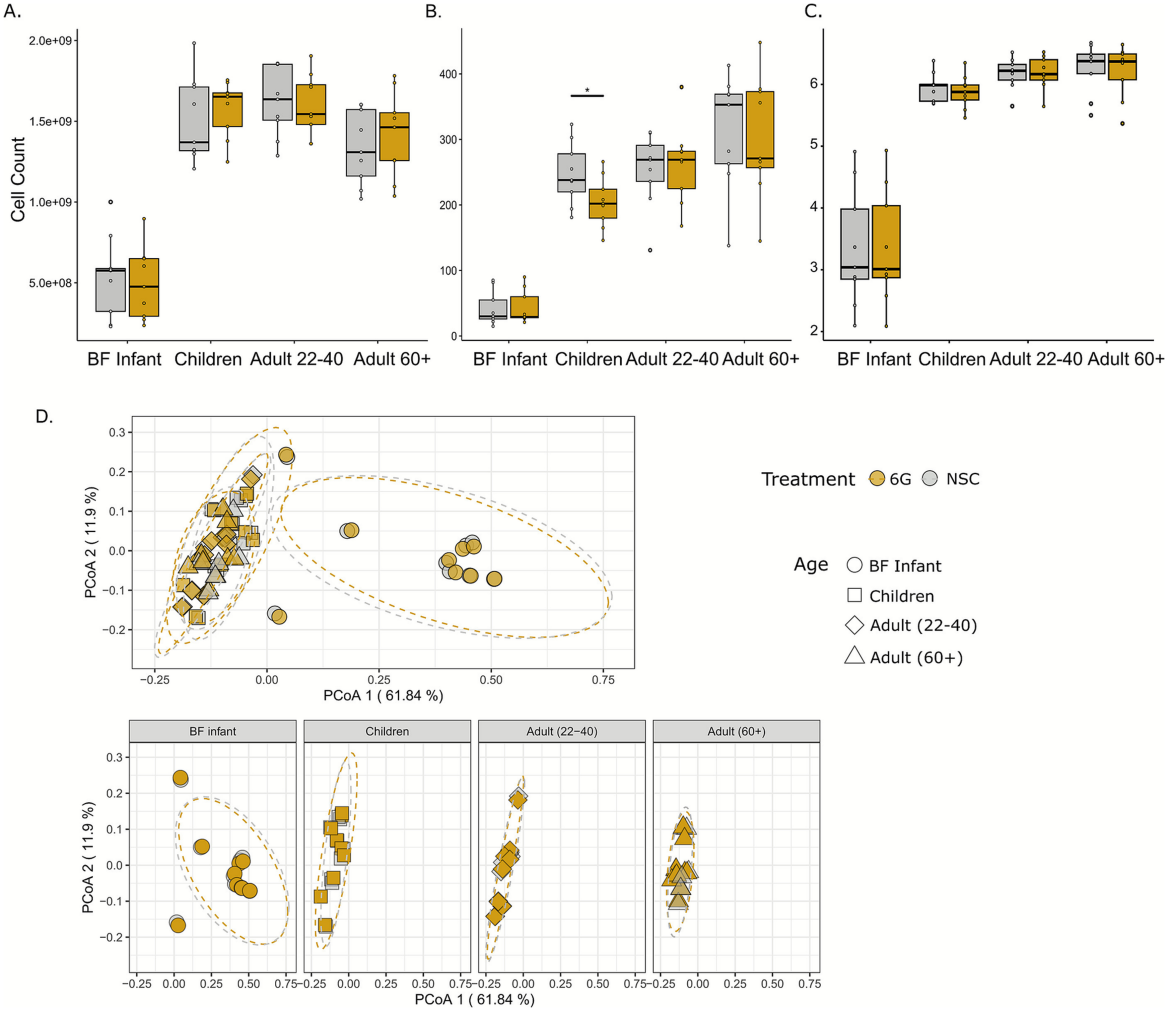
Gut microbiome community structure based on alpha and beta diversity metrics. 6-gingerol groups are shown in dark gold, and non-substrate controls are shown in grey. **(A)** Cell count determined by flow cytometry, **(B)** observed features, **(C)** Shannon’s Index, **(D)** weighted UniFrac analysis with all age groups together (top) and separated (bottom). Significance was determined for Panels **A–C** using paired *t*-tests with Benjamini Hochberg correction. Significance was determined for Panel **D** using PERMANOVA analysis.

Cell counts were performed using flow cytometry to determine community density and are illustrated in Figure 1A. Consistent with previous literature, there was an age-associated increase in cell count after infancy, as demonstrated by an increase in the density of the gut microbiota in children. The cell density remained the same for adults 22–40 and started to decline for adults 60+. Treatment with 6-gingerol did not significantly impact the density of any of the age groups. Next, two alpha diversity measures were employed to determine whether the intra-sample structure of the gut microbiomes changed with 6-gingerol treatment. The alpha diversity metrics used for these purposes were observed features, and Shannon’s Index, shown in Figures 1B,C. Similarly to what was found with community density, alpha diversity was also lowest for the BF infants, with a significant increase (adjusted *p* < 0.05) in alpha diversity for the children group. The increase in alpha diversity continued throughout both adult groups, but increases were not significant. Treatment with 6-gingerol only significantly impacted the children age group (adj. *p* < 0.05) for observed features (Figure 1B). Shannon’s index alpha diversity measures were not impacted significantly by 6-gingerol treatment.

Beta diversity analysis using weighted UniFrac distances and principle coordinates analysis (PCoA) was performed to determine inter-sample diversity. Weighted UniFrac analysis was performed to consider the identity and abundance of the taxa that are shared between samples and is shown in Figure 1D. Similarly to alpha diversity, BF infant samples clustered together, and the other three age groups clustered together, and there was no significant clustering associated with 6-gingerol treatment (PERMANOVA q >> 0.05, see Supplementary Table 1).

**Figure 2:**
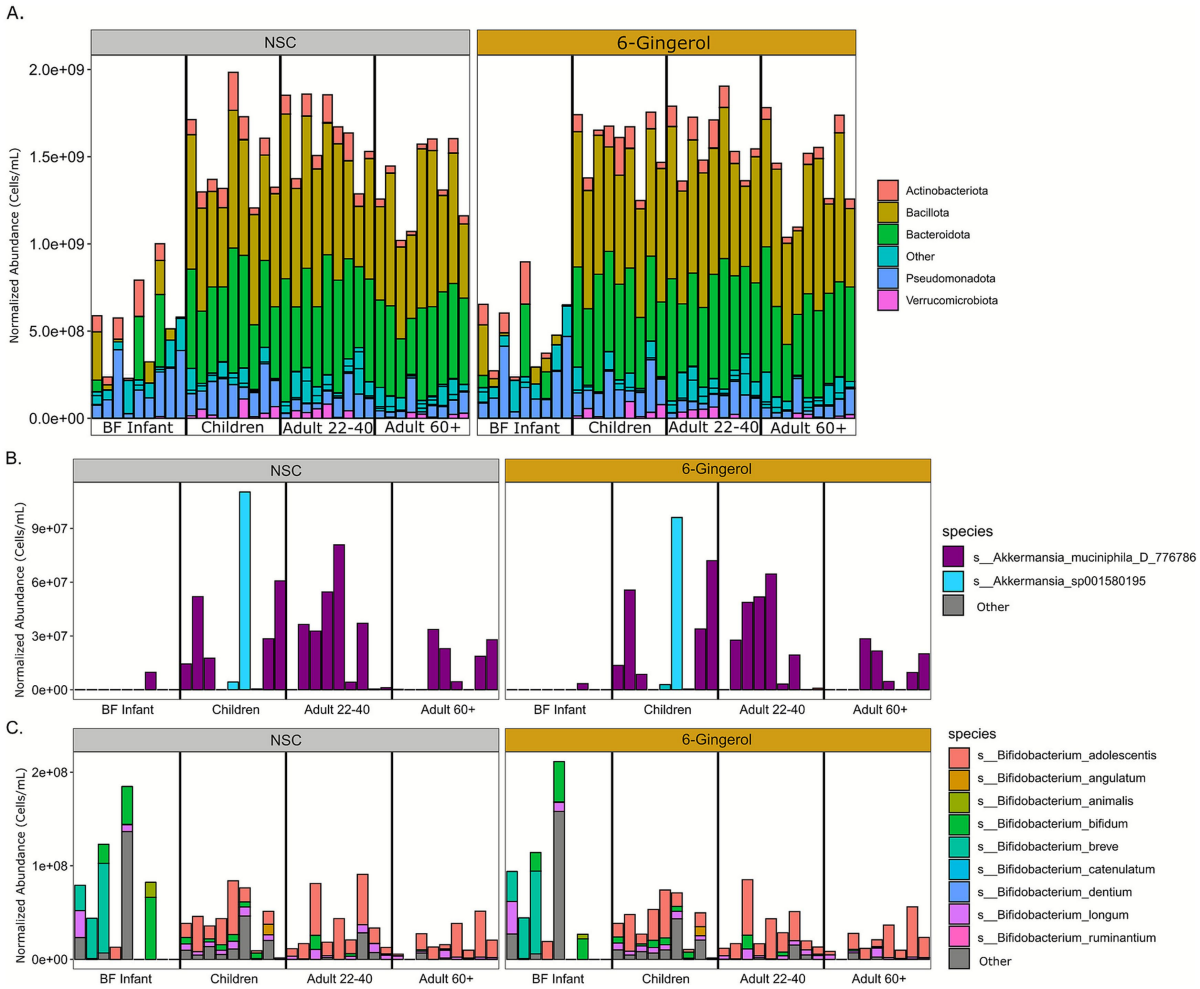
Taxon abundance analysis normalized to cell count data. **(A)** Phylum relative abundance, **(B)** *Akkermansia* abundance, **(C)** *Bifidobacterium* abundance.

Next, bacterial abundance was normalized to cell count data to determine whether there were any changes in taxa abundance with 6-gingerol treatment. At the phylum level, as shown in Figure 2A, there were no significant changes in any age group due to 6-gingerol treatment. Rather, there was a continuation of the expected pattern that the gut microbiota reaches structural stability early in childhood (1). This expected pattern includes the development of communities that are dominated by Bacteriodota and Firmicutes (Bacilota) phyla. Statistical analysis determined no significant changes with respect to 6-gingerol treatment at any age, or any taxonomic level. Previous studies have shown that polyphenols extracted from ginger have increased species within *Akkermansia*, therefore the *Akkermansia* abundance data was extracted and shown in Figure 2B. In this figure, the BF infants had the lowest density of *Akkermansia* present compared with other age groups, but the species that was present was *Akkermansia muciniphila*. Similarly to overall bacterial abundance, *A. muciniphila* increased in abundance for children, where there was also another species present in a few of the donors, *Akkermansia sp001580195*. This species was not present in the adult 22–40 group, and, after childhood, *Akkermansia* decreases in abundance over the course of the age groups. *Bifidobacterium* relative abundance was also extracted for closer exploration due to its known divergence in abundance between age groups (Figure 2C). Here, the data continues to demonstrate expected age progression, as it is shown that the BF infants have the highest abundance of *Bifidobacterium*, as well as the most diversity of species present, whereas throughout the aging process there is a decrease in overall abundance of *Bifidobacterium*, as well as a decrease in diversity. Treatment with 6-gingerol did not change this outcome.

As certain enzymes from the GT1 family (primarily UDP-glycosyltransferases) have been shown capable of glycosylating 6-gingerol, we searched the predicted functional metagenome for genes from this family. There was one possible candidate associated with a member of the *Paraclostridium* genus but this was present in only a single untreated sample at very low read counts. Effectively, the gut microbiomes profiled here do not appear to contain the bacterial functional potential for the degradation of 6-gingerol.

**Figure 3:**
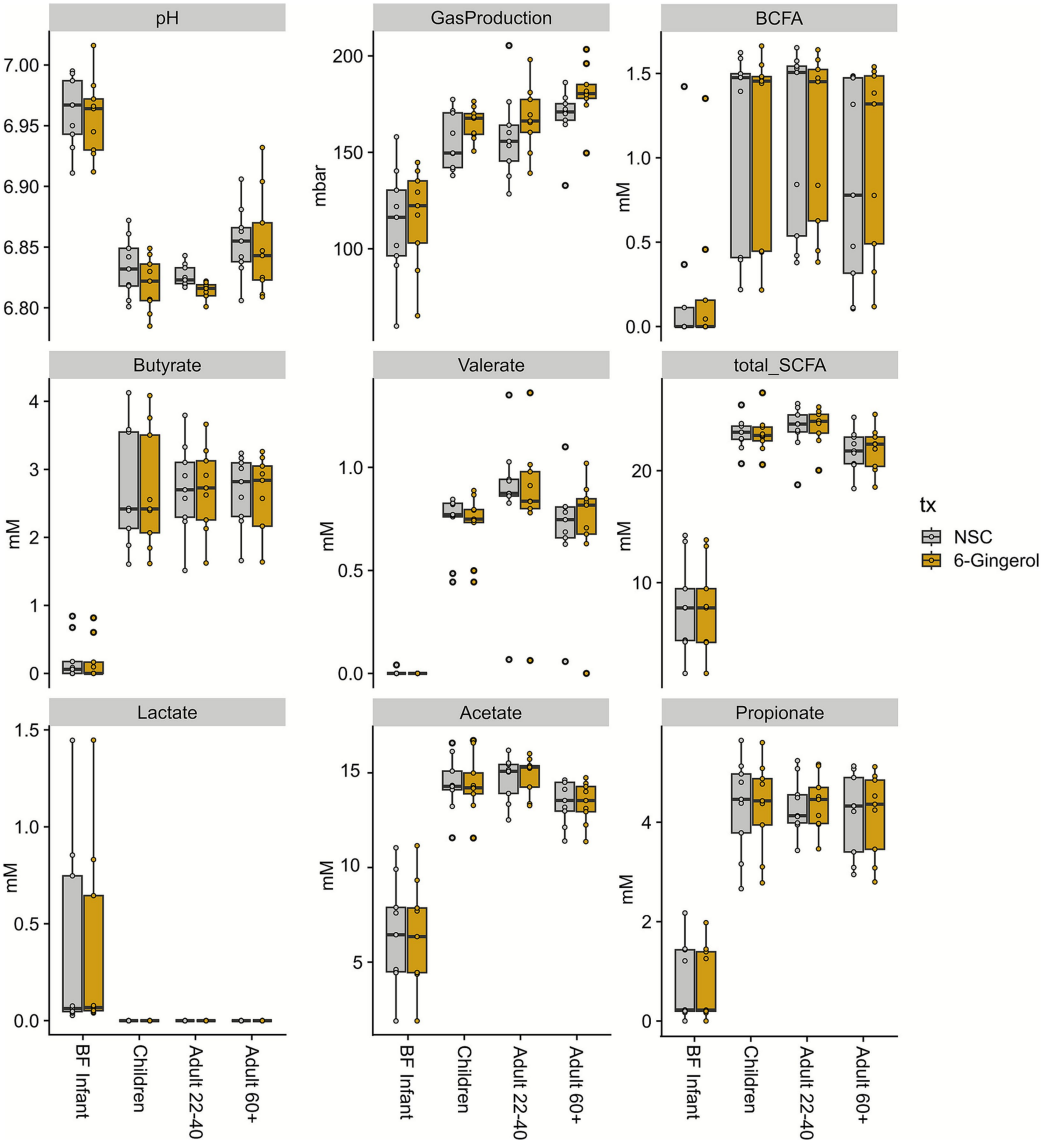
Functional analysis of the microbiome. Statistics were performed using paired *t*-tests and Benjamini Hochberg false discovery rate correction.

Additionally, functional analysis of the gut microbiome was performed by evaluating measures of fermentation, including pH values and gas production (Figure 3). Following 24 h of incubation, the pH of the BF infant group was highest regardless of treatment. As age progressed, there was a decrease in pH for the children and adults 22–40, and then a slight increase for the adults 60+. While there were some variations in pH values with 6-gingerol treatment, none were statistically significant. In terms of gas production, BF infant incubations produced the least gas, and the value increased with the age of the study groups. Once again, while there were slight variations with 6-gingerol treatment, none reached the level of statistical significance. Next, SCFA analysis was performed to determine production of total branched-chain fatty acids (BCFA), SCFAs, and then SCFAs of interest: acetate, butyrate, propionate, and valerate. Lactate was also measured using GC-FID. As is typical, the BF infants had the lowest production of all of these measures except for lactate. Similarly to the results found above, for the rest of the fatty acids, there was an increase in age from BF infants to children, and then the values remain steady for adults 22–40 and adults 60+. As is the case of gas production and pH levels, there was some fluctuation in fatty acid levels with 6-gingerol treatment, however none of those changes were statistically significant.

## Discussion

This study explored the impact of the polyphenol 6-gingerol, one of the main bioactive compounds in ginger on the healthy human gut microbiome across age groups *ex vivo*. Following 24-h incubation, genomic and metabolic analyses were performed to find changes due to 6-gingerol treatment. Overall, this study confirmed previous work that the gut microbiome reaches maturity at early childhood, as expressed by the significant changes found between the BF infants and all other age groups, in all categories of analysis demonstrated here (1, 32).

The mechanism of degradation of 6-gingerol by the gut microbiota has not been thoroughly elucidated. To our knowledge, there is only one known mechanism of modification by a member of the gut microbiota, and that is by *B. subtilis*, through glycosyltransferases, particularly BsUGT489, which transforms 6-gingerol into 5 byproducts of the glycosylation reaction (16). Through a thorough analysis of the current data, however, we found very limited results for either the presence of *B. subtilis* or the presence of GT family type 1. In fact, we found only one potential candidate from a single donor. This indicates that either most of the individual donors in this experiment are lacking the ability to modify 6-gingerol into its byproducts or that there is a lack of literature available on how the gut microbiota degrades 6-gingerol.

6-gingerol only minimally impacted the healthy gut microbiome *ex vivo* at any age group tested here. In fact, the only statistically significant change due to 6-gingerol treatment was a decrease in observed features of the children age group. This was somewhat surprising, given findings by others that 6-gingerol impacts the gut microbiome by increasing microbial diversity, increasing certain beneficial taxa including *A. muciniphila*, and increasing the production of SCFAs (14, 15, 33, 34). However, most of the studies available use mouse models of disease, such as obesity models with high-fat diets or colitis models. Taken together, these results may indicate that 6-gingerol has an ability to positively influence gut health of those with conditions that include inflammation. The study by Wang et al. (34) demonstrated that 6-gingerol decreased inflammatory factors, including TNF-*α*, LPS, and IL-6. Since this study design does not include mammalian tissue, our results are limited in only determining impacts on the gut microbiota alone. Therefore, our results indicate that 6-gingerol has limited impact on the gut microbiota of healthy individuals at the dose of 1.2 mg/L. This is in line with previous results from our group exploring the impact of different polyphenols extracts of spices that are often used for their health benefits with respect to gut health, such as cinnamon, turmeric, and cumin (35). Other work performed by our group to look at the impact of extracts known to have harmful effects on the gut microbiota, such as Senna seed has shown drastic changes to the gut microbiota over a similar time frame (17). Taken together, this may be an indication that polyphenols that benefit the health of the gut microbiota could do so by providing a stabilizing effect on a healthy gut microbiota as opposed to an induction of major changes to the gut microbiota structure or function, however this assertion requires more detailed study.

## Data Availability

The original contributions presented in the study are publicly available. This data can be found at the NCBI Sequence Read Archive with accession number: PRJNA1372016, https://www.ncbi.nlm.nih.gov/sra/?term=PRJNA1372016.
